# Insulin edema after initiation of hybrid closed-loop insulin pump therapy with continuous glucose monitoring: a case report

**DOI:** 10.1186/s40842-022-00143-0

**Published:** 2022-09-30

**Authors:** Mostafa Vasigh, Rachel Hopkins

**Affiliations:** 1 Department of Medicine, State University of New York Upstate Medical University, Syracuse, USA; 2 Department of Medicine, Division of Endocrinology, State University of New York Upstate Medical University, 750 East Adams Street, Syracuse, NY 13210 USA

**Keywords:** Insulin edema, Hybrid closed-loop insulin pump, Type 1 diabetes mellitus, Continuous glucose monitor

## Abstract

**Background:**

Insulin edema is a rare complication which can present after initiation or intensification of insulin therapy in people with diabetes. Initiation of closed-loop hybrid insulin pump therapy can result in rapid improvement in glycemic control for people with diabetes. We present a case in which transition to a closed-loop hybrid insulin pump system, followed by significant improvement in glycemic control, led to development of insulin edema in a person with type 1 diabetes.

**Case presentation:**

We present a 51-year-old woman with type 1 diabetes of 16 years duration, on insulin pump therapy for more than 10 years, who presented for follow-up 7 weeks after transitioning to a hybrid closed-loop insulin pump system with continuous glucose monitoring (CGM). She complained of weight gain and bilateral lower extremity edema which had started two weeks after the change in pump modality. Laboratory studies and echocardiogram did not reveal any etiology of the acute edema. HbA1c was 3.3% lower than the previous measurement 15 weeks earlier, and there was a significant increase in the daily total insulin dose. With exclusion of other causes of acute edema, the patient was diagnosed with insulin edema and started on hydrochlorothiazide. On follow up, her lower extremity edema significantly improved although her weight did not return to baseline.

**Conclusion:**

To our knowledge, this is the first case of insulin edema reported in a person with type 1 diabetes using CGM and a hybrid closed-loop insulin pump system. The increase in total daily insulin dose, rapid improvement of glycemic control, and lack of hypoglycemic episodes were important factors to consider in evaluation of this case. Use of hybrid closed-loop systems can help achieve rapid improvement in glycemic control in people with diabetes. This case suggests that consideration should be given to adjusting initial blood glucose targets when starting these remarkable new technologies in people with baseline poor glycemic control.

## Background

Insulin edema rarely occurs with initiation or intensification of insulin treatment in people with diabetes. It can manifest as lower extremity edema or, less commonly, as generalized edema [1–3], and may be overlooked in the differential diagnosis of edema. There are no formal diagnostic criteria for insulin edema; the entity is therefore a diagnosis of exclusion based on clinical features after ruling out cardiac, renal, and hepatic causes of edema. Here we report a case of insulin edema in an adult who had recently transitioned from insulin pump therapy without a CGM to the use of a hybrid closed-loop insulin pump system. We believe this is the first report of insulin edema in an individual with type 1 diabetes occurring after transitioning to a hybrid closed-loop system.

## Case presentation

This woman is 51 years old, with type 1 diabetes of 16 years duration, and a history of hypertension, obesity, obstructive sleep apnea, and hypothyroidism. She had been using an insulin pump for more than 10 years, most recently with U-200 lispro insulin. She presented to her endocrinologist for a follow-up visit with complaints of weight gain and bilateral lower extremity edema which had started about two weeks after a change in her insulin delivery system. Seven weeks before this visit she had transitioned from using the Accu-Chek Spirit Combo insulin pump without CGM to the Medtronic 670G with the Guardian sensor which she was using in Auto Mode.

She had a history of poor glycemic control with HbA1c levels continuously above 9.4% over the past 10 years. At her last office visit before the pump upgrade, she had a HbA1c of 11.7%. A change in insulin pump technology was recommended in hopes of improving her glycemic control (Table 1). When she subsequently transitioned to the Medtronic 670G pump with the Guardian sensor, she continued the use of insulin U-200 lispro and had automated basal insulin delivery (Auto Mode feature) enabled.

**Table 1:** Body weight, HbA1c, insulin concentration, and average total daily insulin administration

**Timeline**	Insulin Type	Hemoglobin A1c (%)	Body Weight (Kg)	Mean Total Daily Insulin Dose (units/day)
Preceding Year	Lispro U-200			80–90
Week -8^a^	Lispro U-200	11.7	100.7	—
Week 0	Lispro U-200	—	—	—
Week 7	Lispro U-200	8.4	108.3	148
Week 19	Lispro U-200	7.5	109.7	152
Week 31	Lispro U-200	—	109.3	166

^a^8 weeks before initiation of hybrid closed-loop system

In addition to insulin, her medications at week 0 (Auto mode started) were aspirin, atorvastatin, calcium carbonate, esomeprazole, levothyroxine, lisinopril, norethindrone, and cholecalciferol with occasional use of ibuprofen as needed.

At her week 7 visit, her weight was over 7 kg higher than baseline (Table 1). Physical exam revealed pitting edema in the lower extremities bilaterally up to the mid-shin. No abnormal lung sounds or jugular venous distension was noted. Further evaluation included a comprehensive metabolic panel (creatinine 0.81 mg/dL, albumin 3.6 g/dL, ALT 12 U/L), TSH (normal), ACTH and cortisol (normal), and an echocardiogram that did not show any evidence of congestive heart failure or abnormalities that could be associated with weight gain or edema. She had preexisting albuminuria with a urine albumin of 593.1 mg/L seven months prior and 166.8 mg/L at the time of presentation with edema. With a normal serum albumin level (as reported above) she did not meet diagnostic criteria for nephrotic syndrome.

Point-of-care testing at the visit revealed a HbA1c of 8.4% which was a reduction of 3.3%, and the lowest HbA1c recorded over the previous 10 years. Insulin pump data showed that she was using Auto Mode 98% of the time and had been receiving an average of 152 units of insulin daily for the 27 days prior to the office visit. Glucose time in range (70–180 mg/dL) was 85% with 15% of the time in the 180–250 mg/dL range and no hypoglycemia. Mean blood glucose was 146 mg/dL. For comparison, her mean blood glucose on her glucometer download at the visit 4 months earlier was 281 mg/dL (SD 106 mg/dL) based on average testing rate of 3.1 readings per day. Review of pump download data for the previous year shows that the total daily insulin doses were between 80 and 90 units a day. Therefore, the total daily insulin dose on the hybrid closed-loop system was significantly higher (Table 1).

With the exclusion of other causes of acute edema, the patient was diagnosed with insulin edema and started on hydrochlorothiazide by her primary care provider. At follow-up visits with her endocrinologist, her HbA1c continued to improve, with an A1c of 7.5% at week 19. Her lower extremity edema resolved and her weight stabilized, although she failed to lose any of the weight that she gained upon initiation of the hybrid closed-loop system. Her urine albumin levels remain elevated after resolution of edema; she was seen by a nephrologist who diagnosed diabetic nephropathy.

## Discussion and conclusions

Insulin edema was first described by Aaron Leifer in 1928 [4]. Although minor insulin edema might be underrecognized, the incidence of notable or severe insulin edema seems to be a rare event [1]. Several clinical features have been described as risk factors for developing insulin edema including poor glycemic control, new-onset diabetes, type 1 diabetes, low body weight, poor nutritional status, and higher doses of insulin therapy [1, 5]. Many reported cases of insulin edema have been in substantially malnourished or underweight individuals [3, 5, 6]. Our patient has type 1 diabetes, many years of poor glycemic control, and underwent recent intensification of insulin therapy resulting in relatively high daily insulin doses. However, unlike many reported cases, our patient had baseline obesity and presumably adequate nutritional status before developing edema. This bears some resemblance to a case described in 2015 in which the patient had obesity, was not insulin naïve, and developed insulin edema a few days after commencing insulin pump therapy, which resulted in a dramatic and abrupt improvement of glycemic control [7]. We also recently reported insulin edema in an adult with type 2 diabetes using automated basal insulin delivery of U-500 insulin (total daily insulin delivery of > 500 units daily; A1c 8.3%) [8]. In contrast, the patient in this report has type 1 diabetes, had significantly lower insulin requirements and achieved better glycemic control. The 2 cases have in common use of a concentrated insulin in their pump and experienced a rapid significant improvement in glycemic control.

As noted above, insulin edema is a diagnosed of exclusion. In our patient the most common causes of edema, congestive heart failure and hypoalbuminunemia, were excluded leading to our diagnosis of insulin edema. The pathogenesis of insulin edema is not known. The most commonly proposed mechanisms relevant to our patient are the antinatriuretic effect of insulin and increased capillary permeability associated with chronic hyperglycemia [1, 2]. Additional proposed mechanisms relate to fluid resuscitation (during treatment of severe hyperglycemia or diabetic ketoacidosis) and increased glucocorticoid production due to insulin-induced hypoglycemia. Neither of these would apply to our patient; she did not undergo any aggressive fluid treatment and the use of CGM allowed us to confirm that hypoglycemia was not a contributing factor.

Most cases of insulin edema have been relatively benign and self-limited. Even uncomplicated edema is an undesirable occurrence and in one case report, recurrent insulin-induced edema seems to have contributed to nonadherence to insulin regimen in a young patient with cystic fibrosis-related diabetes [9]. However, cases have been reported in which insulin edema precipitated more serious events such as pulmonary edema, congestive heart failure, and acute renal failure [10–13] even in the absence of predisposing medical conditions.

There is no clear standard for treatment of insulin edema. Our patient’s condition was treated with diuretic therapy and appears to have been responsive, although the literature suggests that the edema would likely have responded to dietary sodium restriction and might also have simply resolved on its own over time [14]. Ephedrine has been used with success in severe recurrent insulin edema [15].

In this case, insulin delivery increased significantly after initiation of hybrid closed-loop therapy with an approximately 75% increase in total daily insulin dose. Although basal insulin delivery often decreases with use of hybrid closed-loop systems compared to traditional insulin pump therapy [16], in the presence of elevated HbA1c, initiation of these systems can result in higher basal insulin delivery and higher insulin delivery overall. It would be expected that adults with poor glycemic control, who are underinsulinized will experience an increase in overall dose with hybrid closed-loop systems.

The weight gain experienced by this woman remained even after resolution of edema. Weight gain is a common side effect of initiation or intensification of insulin treatment and although the exact mechanism is not known, both fat accumulation and fluid retention are thought to be involved.

In our case, the use of concentrated insulin U-200 lispro in the insulin pump is off-label. We do not believe this contributed substantially to the development of her insulin edema. First, the patient had already been using U-200 lispro for several years in her insulin pump prior to transitioning to the hybrid closed-loop system and second, U-200 lispro has been determined to be bioequivalent with pharmacokinetics similar to rapid-acting U-100 insulins [17, 18]. Presumably, her insulin requirements would be the same with either concentration of insulin, although it is possible that higher doses were reached more rapidly using the U-200 formulation. The use of concentrated insulins in hybrid closed loop systems warrants further study.

To our knowledge, this is the first report of insulin edema in a person with type 1 diabetes using CGM and a hybrid closed-loop system. This allows a closer inspection of the glycemic variables present with the development of this rare complication and makes clear that hypoglycemia was not a factor in her development of insulin edema. This case also points to a potential concern about initiation of these systems in patients with poor glycemic control at baseline. Overly-rapid improvement in glycemic control has long been known to increase the risk of decompensating diabetic retinopathy. This rare case of insulin edema reminds us of the potential risks of rapid reductions in HbA1c that can be associated with the use of these remarkable new technologies. Consideration might be given to adjusting blood glucose targets when initiating hybrid closed-loop insulin pump therapy in people with baseline poor glycemic control. Given the increasing use of automated insulin delivery systems in type 1 diabetes, future studies examining the best approaches for initiating this therapy in people with high insulin requirements should be considered.

## Data Availability

The datasets used and/or analysed during the current study are available from the corresponding author on reasonable request.

## Abbreviations

CGM: Continuous glucose monitor
